# Antigenic Site-Specific Competitive Antibody Responses to the Fusion Protein of Respiratory Syncytial Virus Were Associated With Viral Clearance in Hematopoietic Cell Transplantation Adults

**DOI:** 10.3389/fimmu.2019.00706

**Published:** 2019-03-29

**Authors:** Xunyan Ye, Obinna P. Iwuchukwu, Vasanthi Avadhanula, Letisha O. Aideyan, Trevor J. McBride, Laura L. Ferlic-Stark, Kirtida D. Patel, Felipe-Andres Piedra, Dimpy P. Shah, Roy F. Chemaly, Pedro A. Piedra

**Affiliations:** ^1^Department of Molecular Virology and Microbiology, Baylor College of Medicine, Houston, TX, United States; ^2^Department of Epidemiology and Biostatistics, The University of Texas Health Science Center at San Antonio, San Antonio, TX, United States; ^3^Departments of Infectious Diseases, Infection Control and Employee Health, The University of Texas MD Anderson Cancer Center, Houston, TX, United States; ^4^Department of Pediatrics, Baylor College of Medicine, Houston, TX, United States

**Keywords:** competitive antibody, fusion protein, viral clearance, respiratory syncytial virus, hematopoietic cell transplantation

## Abstract

**Background:** Recent studies of human sera showed that the majority of the respiratory syncytial virus (RSV) neutralizing antibodies are directed against pre-fusion conformation of the fusion (F) protein of RSV and revealed the importance of pre-fusion antigenic site Ø specific antibodies. However, detailed analysis of multiple antigenic site-specific competitive antibody responses to RSV F protein and their contribution to virus clearance in humans are lacking.

**Methods:** We prospectively enrolled a cohort of RSV infected hematopoietic cell transplantation (HCT) adults (*n* = 40). Serum samples were collected at enrollment (acute, *n* = 40) and 14 to 60 days post-enrollment (convalescent, *n* = 40). Antigenic site-specific F protein antibodies were measured against pre-fusion site Ø, post-fusion site I, and sites II and IV present in both the pre-fusion and post-fusion F protein conformations utilizing four different competitive antibody assays developed with biotinylated monoclonal antibodies (mAb) D25, 131-2A, palivizumab, and 101F, respectively. The lower limit of detection were 7.8 and 1.0 μg/mL for the competitive antibody assays that measured site Ø specific response, as well as sites I, II, and IV specific responses, respectively. Neutralizing antibody titers to RSV A and B subgroups was determined by microneutralization assays.

**Results:** The overall findings in RSV infected HCT adults revealed: (1) a significant increase in antigenic site-specific competitive antibodies in convalescent sera except for site Ø competitive antibody (*p* < 0.01); (2) comparable concentrations in the acute and convalescent serum samples of antigenic site-specific competitive antibodies between RSV/A and RSV/B infected HCT adults (*p* > 0.05); (3) significantly increased concentrations of the antigenic site-specific competitive antibodies in HCT adults who had genomic RSV detected in the upper respiratory tract for <14 days compared to those for ≥14 days (*p* < 0.01); and (4) statistically significant correlation between the antigenic site-specific competitive antibody concentrations and neutralizing antibody titers against RSV/A and RSV/B (r ranged from 0.33 to 0.83 for acute sera, and 0.50–0.88 for convalescent sera; *p* < 0.05).

**Conclusions:** In RSV infected HCT adults, antigenic site-specific antibody responses were induced against multiple antigenic sites found in both the pre-fusion and post-fusion F conformations, and were associated with a more rapid viral clearance and neutralizing antibody activity. However, the association is not necessarily the cause and the consequence.

## Introduction

Respiratory syncytial virus (RSV) is a negative-sense, single-stranded RNA virus of the family *Pneumoviridae*. RSV is transmitted via fomites and large droplet aerosols. RSV, traditionally considered as the most common respiratory pathogen in children <5 years old, has more recently been found to have a high prevalence in immunocompromised individuals (1, 2). RSV is also a leading cause of severe respiratory infection in children and adults with a compromised immune system, with mortality rates as high as 80% (3). The hematopoietic cell transplantation (HCT) recipients are highly vulnerable to the severe consequences of RSV infection with the highest risk for mortality within 100 days after transplantation (4). A licensed vaccine against RSV is not yet available because of problems with the stability, purity, reproducibility, tolerability, and potency of vaccine candidates (5–8). Moreover, immunocompromised patients may not respond adequately to vaccinations because of their relative immunologic suppression. Current treatment strategies of demonstrated efficacy in immunocompromised patients include antiviral therapies such as ribavirin, palivizumab, and immunomodulation with total IVIG (9–11).

RSV fusion (F) surface glycoprotein is a class I protein that mediates viral entry into host cells by transforming from a metastable trimeric pre-fusion conformation (pre-F) to a highly stable post-fusion conformation (post-F). The antigenic topology of RSV F is substantially altered during this transition. Molecules that prevent these structural changes can prevent viral fusion and have potential as therapeutics for treatment of RSV infection. Some groups of epitopes, referred to as antigenic sites, are generally conserved on both the pre-F and post-F conformations, whereas others are found either on the pre-F or post-F conformation (12). Six antigenic sites on the F protein have been described: Ø-V. Antigenic site Ø (“zero”) presents at the apex of the pre-F trimer. The pre-F-specific antibodies (MEDI8897 and D25) recognize antigenic site Ø (13). Antigenic site I is found in the post-F conformation. mAb 131-2A binds to antigenic site I on the post-F confirmation (14, 15). Antigenic sites II and IV are found on both pre-F and post-F conformations. Palivizumab (Synagis) is the first characterized monoclonal antibody (mAb) and currently the only mAb licensed for the prevention of severe RSV infection in high-risk infants. It recognizes antigenic site II. mAb 101F recognizes antigenic site IV. Although the secondary structure elements that form site III are present on both pre-F and post-F, they adopt a different spatial arrangement in post-F that results in higher-affinity binding to pre-F (16). The potently neutralizing mAb MPE8 was shown to recognize antigenic site III (16). Antigenic site V is found in the pre-F conformation and located between sites Ø and III. mAb AM14 targets antigenic site V and is a potently neutralizing mAb (17, 18).

RSV is primarily a mucosally restricted virus causing damage to the upper and lower respiratory tracts during its multiple rounds of replication. It can elicit cellular and humoral immune responses at variable levels in HCT recipients, depending upon the degree to which individuals remain immunocompromised post-transplantation. RSV-specific neutralizing antibody play an important role in preventing severe infection while cellular immune responses are thought to play a critical function in destroying RSV infected cells and thus clearing the infection. Previously RSV naïve but immunocompetent infants can shed RSV from their upper respiratory tract (URT) for up to 21 days; by contrast, immunocompromised children can shed RSV for several months (19, 20). In the murine model of RSV infection, CD8+ T cells are capable of clearing RSV infection (14, 21–23). Limited data from studies of infants with primary RSV infection suggest that a cellular immune response with specific cytotoxic T lymphocytes are initiated within 10 days of infection (24, 25). Studies on the humoral immune responses after primary RSV infection in children and re-infection in children and adults have been reported (26–28). Although administration of intravenous immunoglobulin (IVIG) with high neutralizing antibody activity against RSV and palivizumab provides protection against severe RSV infection in high-risk infants, the role of neutralizing antibodies in recovery from an established infection is not clear. We have previously demonstrated that significantly greater levels of humoral palivizumab-competing antibody and neutralizing antibody titers are associated with more rapid RSV clearance in HCT recipients. This cohort of forty RSV infected HCT adults provided us an opportunity to further study other RSV epitope-specific competitive antibody levels and identify additional immune correlates of viral clearance. Three more RSV epitope-specific competitive antibody assays were developed and standardized to detect competitive antibodies to antigenic sites Ø, I, and IV. In addition, understanding the relationship between neutralization antibody and epitope specificity of the competitive antibodies elicited in response to natural RSV infection will be critical in the selection and design of new monoclonal antibodies and vaccines for prevention of RSV.

## Methods

### Study Subjects

HCT recipients with laboratory-confirmed RSV upper respiratory tract infection at enrollment and negative chest radiography findings were enrolled within 72 h of RSV diagnosis and stratified by level of risk for progression to the lower respiratory tract as previously described (29). From January 2012 to April 2015, sera were collected from all patients at enrollment (acute) and 14–60 days after hospitalization (convalescent) for evaluating the humoral immune response. Nasal wash samples were collected at enrollment, day 7 (±1), day 14 (±1), and between day 21 and day 28 (±1) for detecting of viruses in the RSV/A and RSV/B subtypes by real-time, reverse-transcription polymerase chain reaction (rtRT-PCR). At enrollment, an interview was performed to obtain historical information, and medical records were reviewed to extract demographic and clinical data. The institutional review boards of the University of Texas MD Anderson Cancer Center and Baylor College of Medicine approved the study protocol and written informed consent was obtained from all the participants.

### Real-Time Reverse-Transcription Polymerase Chain Reaction (rtRT-PCR)

Viral RNA extraction and RSV/A and RSV/B detection in nasal wash samples by rtRT-PCR in a CLIA certified Respiratory Virus Diagnostic laboratory (CLIA ID# 45D0919666) were performed as previously described (30).

### Antigenic-Site Competitive Antibody Assays

Four antigenic-site competitive antibody assays were used to measure concentrations of D25-competing antibody (site Ø), 131-2A-competing antibody (site I), palivizumab-competing antibody (site II), and 101F-competing antibody (site IV) in serum that compete with biotinylated mAbs for binding to the respective antigenic site of the RSV fusion protein. D25 and 101F were purchased from Cambridge Biologics, LLC, Brookline, MA, USA. Palivizumab was from MedImmune, LLC, Gaithersburg, MD, USA. 131-2A was from EMD Millipore Corporation, Temecula, CA, USA. mAbs were biotinylated with a Pierce^TM^ Antibody Biotinylation Kit (Pierce, Rockford, IL, USA) per manufacturer's instructions. The source of the fusion protein was from sucrose purified RSV/A/Bernett (spRSV, GA1) for sites I, II and IV. One hundred μL of spRSV (total protein 7 μg/mL) was coated onto the Immulon 2HB 96-well plate (Thermo Scientific, Waltham, MA, USA) for 18 h at 4°C. For site Ø competitive antibody assay, 100 μL of HEp-2 cell suspension (24 x 10^4^ cells /mL) in 10% FBS/MEM were seeded on 96-well Falcon tissue culture plates from Corning (Pittston, Pennsylvania, USA). The plates were incubated at 37°C with 5% CO_2_ and 85% humidity for 24 h to form monolayers. Cells were inoculated with spRSV (MOI = 0.002) and incubated at the same condition for 2 days. The plates from all four assays were blocked for 1 h with 5% milk (Carnation Instant Nonfat Dry Milk) in 1X phosphate-buffered saline. One hundred μL of D25, 131-2A, palivizumab, and 101F at a concentration of 1.25 μg/mL in 5% milk (for 131-2A, palivizumab, and 101F) and 5% milk in 10% Defined Fetal Bovine Serum in Minimum Essential Medium (for D25) were added in duplicate followed by 2-fold serial dilutions (12,500 to 24.41 ng/mL) for generating a standard curve on each plate. Next 50 μL of 2-fold serial dilutions of serum samples (1:5 to 1:2,560) in duplicate were added to the coated plates, and then immediately 50 μL of 100 ng/mL of biotinylated mAbs (D25, 131-2A, palivizumab, and 101F) generated with a Pierce^TM^ Antibody Biotinylation Kit (Pierce, Rockford, IL) per manufacturer's instructions, were added, followed by 1 h incubation. After washing, HRP-conjugated streptavidin (SeraCare Life Sciences, Gaithersburg, MD) was added for an additional hour. Wells containing biotinylated mAb without sera served as positive controls representing maximum binding. Wells without biotinylated mAbs containing either 5% milk instead of sera or wells containing sera served as negative controls. A four-parameter logistic (4PL) regression model was used to calculate the competitive antibody concentrations (μg/mL) based on the dynamic range of the standard curve by interpolating the concentration of the standards that corresponds to the absorbance value at which the test serum sample resulted in 50% inhibition. The lower limit of detection (LLoD) was 1.0 μg/mL for site I, II, and IV competitive antibody assays, and 7.8 ug/mL for site Ø competitive antibody assay. Samples with concentration below the LLoD were assigned a value of 0.5 and 3.9 μg /mL, respectively.

### RSV F Protein Specific Microneutralization (MN) Assay

Serum samples and the four mAbs (D25, 131-2A, palivizumab, and 101F) were analyzed for neutralizing antibodies (Nt Ab) against RSV/A/Tracy and RSV/B/18537 in HEp-2 cells using a qualified microneutralization assay as previously described (31–33). Serum samples and mAbs (final concentration was 40 μg/mL) were diluted 1:8 initially, followed by 2-fold serially dilution. An equal volume of RSV/A Tracy or RSV/B 18537 was added to each dilution and incubated at 36°C with 5% CO_2_ for 90 minutes. One hundred μL of HEp 2 cells was added to each well in the 96-well plate and incubated for 6–7 or 7–8 days for RSV/A and RSV/B microneutralization assays, respectively. Then the 96-well plates were fixed and stained with 10% formalin/0.01% crystal violet solution for ~24 h. After the 96-well plates were air-dried, they were read. Neutralizing antibody titers were defined as the final dilution at which there was a 50% reduction in viral cytopathic effect (CPE). Any serum sample resulting in a titer < LLoD (2.5 log2) was assigned a value of 2 log2. Any mAbs resulting in a titer < LLoD was retested using the stock (1.0 mg/mL) and assigned a value of 0.2 log2 if the titer was negative again.

### Statistical Analysis

For demographic characteristics and clinical outcomes, continuous variables and categorical variables were analyzed. A paired *t*-test was used to determine whether the geometric means of neutralizing antibody titer (GMT) (log2) or geometric mean of log transformed competitive antibody concentrations (GMC) differed significantly between acute and convalescent samples. A two-sample *t*-test was used to determine whether the neutralizing antibody (log2) or GMC of log transformed competitive antibody concentrations differed significantly between RSV/A and RSV/B infected patients, as well as between HCT recipients who shed virus for < 14 and ≥ 14 days. Statistical significance was indicated for *p*-values < 0.05. Pearson's correlation coefficients were calculated between neutralizing antibody titers and site-specific competitive antibody concentrations. Statistical analyses were performed using the SPSS Statistic 22 (IBM, Armonk, NY).

## Results

### Demographic and Clinical Variables of RSV Infected HCT Adults Were Comparable Between Study Groups

Clinical characteristics at enrollment are summarized for all 40 HCT adults in Table 1 from previous publication (29), stratified by duration of RSV shedding (<14 or ≥14 days) or by RSV infection subtype (RSV/A or RSV/B). Briefly, all listed variables were comparable between the groups. The only significant difference observed was recipients who shed RSV for ≥14 days were more likely to have received an allogenic stem cell transplant compared to recipients with a shorter duration of viral shedding (18/20 vs. 11/20, *p* < 0.025). The absolute neutrophil count and the absolute lymphocyte count are within the normal ranges for these cohorts. The median time from transplantation to RSV infection is 169 and 100 days for adults shedding RSV from the URT for < 14 and ≥14 days, respectively.

**Table 1:** RSV Antibody Levels in Acute and Convalescent Sera from RSV infected HCT Adults.

**RSV antibody**	**Ab target**	**Acute (*n* = 40)**	**Convalescent (*n* = 40)**	**Fold increase**	***P*-value^b^**
Competitive antibody	Antigenic site Ø	21.1 [14.9, 30.6]^a^	55.1 [37.5, 78.0]	2.6	<0.001
	Antigenic site I	2.0 [1.4, 3.0]	8.2 [4.8, 14.7]	4.1	<0.001
	Antigenic site II	2.9 [1.9, 4.4]	16.3 [8.9, 29.8]	5.6	<0.001
	Antigenic site IV	7.0 [5.0, 10.4]	28.2 [18.0, 47.3]	4.0	<0.001
Neutralizing antibody	RSV/A	6.9 [6.3, 7.5]	9.6 [8.7, 10.4]	6.5	<0.001
	RSV/B	7.2 [6.4, 8.1]	9.9 [8.8, 10.9]	6.5	<0.001

a *Geometric mean conc., μg/mL for competitive antibody and log2 for neutralizing antibody titer [95% Confidence Interval]*.

b *Paired t-test for difference in means of competitive antibody conc. (log2 μg/mL) or neutralizing antibody titer (log2) between acute and convalescent*.

### The Site-Specific Competitive Antibody Assays Were Specific to the Corresponding Competitive Antibodies

**Figure 1:**
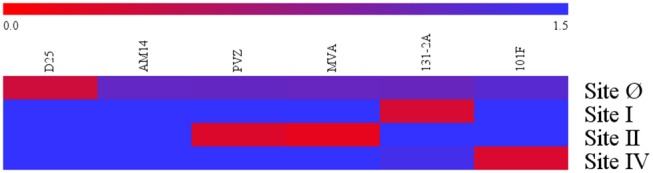
Specificity of RSV antigenic site-specific competitive antibody assays. Blue indicates there was the mAbs had no inhibition to the biotinylated mAbs specific to the RSV antigenic site. Red indicates there were strong inhibition to the RSV antigenic site. The 0.0–1.5 scale represents the level of mAb inhibition from the strongest to the weakest. PVZ, palivizumab; MVA, motavizumab. Data represented are from 3 independent duplicate competitive antibody assays.

Specificity of the four competitive antibody assays were confirmed using a panel of monoclonal antibody (12.5 μg/mL) to compete with biotinylated monoclonal antibody specific to their respective antigenic sites (Figure 1). Using a 125-fold higher concentration, the panel of monoclonal antibodies that bound antigenic sites on the F protein other than the site targeted by the biotinylated monoclonal antibody were not able to inhibit the biotinylated monoclonal antibody from binding to its antigenic site. For example, D25 inhibited or competed with the biotinylated D25 in the site Ø competitive antibody assay, while the rest of the monoclonal antibodies in the panel did not inhibit the biotinylated D25 from binding to site Ø.

### Monoclonal Antibodies Showed Different Levels of Neutralizing Potency

**Figure 2:**
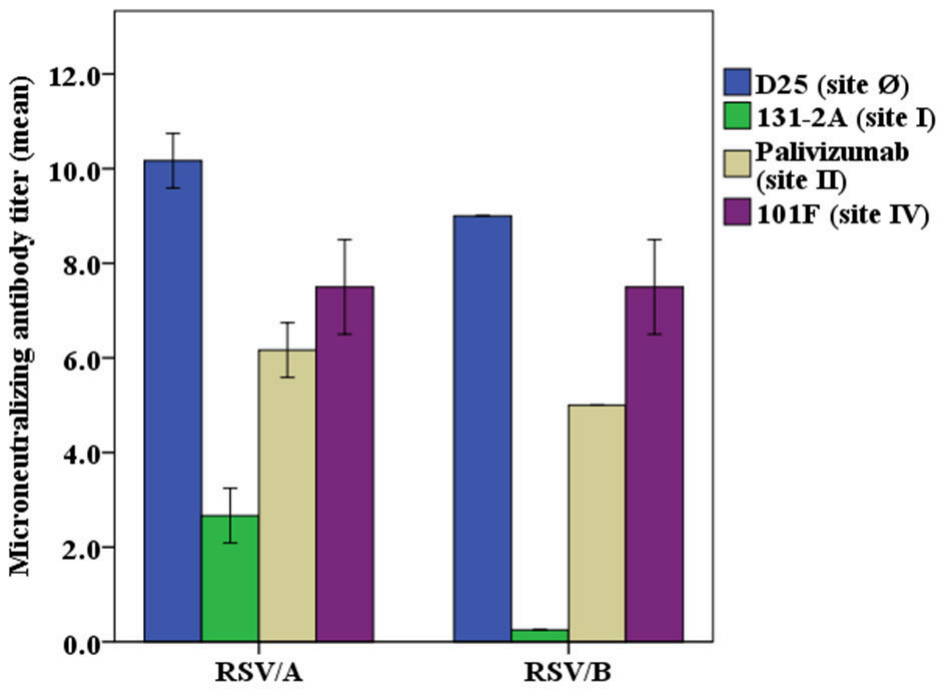
Neutralizing potency of RSV antigenic site-specific monoclonal antibody. RSV/A Tracy and RSV/B 18537 were used in the experiments. The concentration of the mAbs were 40 μg/mL for D25, palivizumab, 131-2A, and 101F in both RSV/A and RSV/B microneutralization assays, except that mAb 131-2A was used at 1.0 mg/mL in the RSV/B microneutralization assay. The Y axis is the RSV/A Tracy or RSV/B 18537 neutralizing antibody titers (log2) for different mAbs used in the microneutralization assay. Data represented are from 3 independent duplicate microneutralization assays ± standard deviation.

Neutralizing activity of the monoclonal antibodies at a concentration of 40 μg/mL was evaluated in the RSV/A and RSV/B microneutralization assays (Figure 2). The mAb D25, that binds antigenic site Ø, is the most potent of the mAbs used in the competitive antibody assays. Palivizumab and 101F mAbs that bind site II and site IV, respectively, have moderate neutralizing antibody activity, and 131-2A, a site I mAb, did not have measurable neutralizing activity, which are consistent with the results previously reported (34).

### Convalescent Sera Had Higher Concentration of Site-Specific Competing or Neutralizing Antibody Than Acute Sera

A significant increase in RSV antibody levels (competitive and neutralizing antibodies) in the convalescent sera compared to the acute sera was measured by all four competitive antibody assays and both microneutralization assays (Table 1). The GMC of D25-competing antibody (Table 1) was the highest in both acute and convalescent sera, followed by the 101F-competing antibody (site IV), palivizumab-competing antibody (site II), and 131-2A-competing (site I) antibodies. The RSV/A and RSV/B neutralizing antibody GMTs were comparable. However, the fold-increase in the competitive antibody concentration was greatest for palivizumab-competing antibody (5.6), and lowest for D25-competing competitive antibodies (2.6). The fold-increase was comparable between RSV/A and RSV/B neutralizing antibody titers (6.5).

### Higher Antibody Levels Were Detected in HCT Adults Shedding RSV <14 Days

The competitive antibody in GMC (μg/mL) and neutralizing antibody in GMT (log2) were compared between HCT adults shedding virus from their URT for <14 and ≥14 days (Table 2). For the acute sera, there was no significant difference between the two groups for either site-specific competitive antibodies or RSV-specific neutralizing antibodies. However, for the convalescent sera, significantly higher levels (<0.01) were detected of both site-specific competitive antibodies and RSV-specific neutralizing antibodies in HCT adults shedding RSV <14 days, except for the site Ø competitive antibody. In addition, all the convalescent sera had higher antibody levels than acute sera for both site-specific competitive antibody and RSV-specific neutralizing antibodies. The total GMC for the four competitive antibodies in the convalescent sera increased about 5-folds for HCT adults shedding virus <14 days and about 2.5 folds for those shedding virus ≥14 days. For HCT adults shedding virus <14 days, the total GMC of the four site-specific antibodies increased from 47.4 μg/mL in the acute sera to 228.6 μg/mL in the convalescent sera. For HCT adults shedding virus ≥14 days, the total GMC increased from 34.1 μg/mL in the acute sera to 91.8 μg/mL in the convalescent sera.

**Table 2:** RSV Antibody Levels between RSV Infected HCT Adults Who Shed RSV <14 days and ≥14 Days.

**RSV antibody**	**Ab target**	**Serum**	**<14 days (*n* = 20)**	**≥14 days (*n* = 20)**	***P*-value^b^**
Competitive antibody	Ø	Acute	22.6 [13.6, 37.2]^a^	26.6 [19.2, 38]	0.712
		Convalescent	59.7 [33.9, 103.8]	50.8 [33.0, 79.1]	0.672
	I	Acute	2.4 [1.3, 5.2]	1.6 [1.0, 2.6]	0.307
		Convalescent	18.2 [7.8, 41.1]	3.7 [2.3, 6.5]	**0.001**
	II	Acute	3.9 [1.9, 8.1]	2.2 [1.4, 3.3]	0.139
		Convalescent	44.4 [18.1, 108.9]	5.9 [3.3, 10.8]	**<0.001**
	IV	Acute	9.0 [5.7, 16.2]	5.5 [3.5, 8.1]	0.156
		Convalescent	60.2 [28.6, 134.0]	13.2 [8.5, 20.6]	**<0.001**
	Ø, I, II, IV	Total Acute	47.4 [29.9, 79.8]	34.1 [22.9, 48.9]	0.306
		Total Convalescent	228.6 [116.8, 400.3]	91.8 [66.6, 123.1]	**0.013**
Neutralizing antibody	RSV/A	Acute	6.97 [6.14, 7.89]	6.22 [5.28, 7.20]	0.281
		Convalescent	10.21 [9.09, 11.37]	8.24 [7.30, 9.29]	**0.016**
	RSV/B	Acute	7.32 [6.30, 8.48]	6.35 [5.35, 7.32]	0.170
		Convalescent	10.78 [9.31, 12.44]	8.01 [6.82, 9.28]	**0.006**

a *Geometric mean conc., μg/mL for competitive antibody and log2 for neutralizing antibody titer [95% Confidence Interval]*.

b *Two-sample t-test for difference in means of antigenic site competitive antibody conc. (log2 μg/mL) or neutralizing antibody titer (log2) between <14 and ≥14 days. The bold values significant means that the p-values are statistically significant at 0.05 level*.

**Figure 3:**
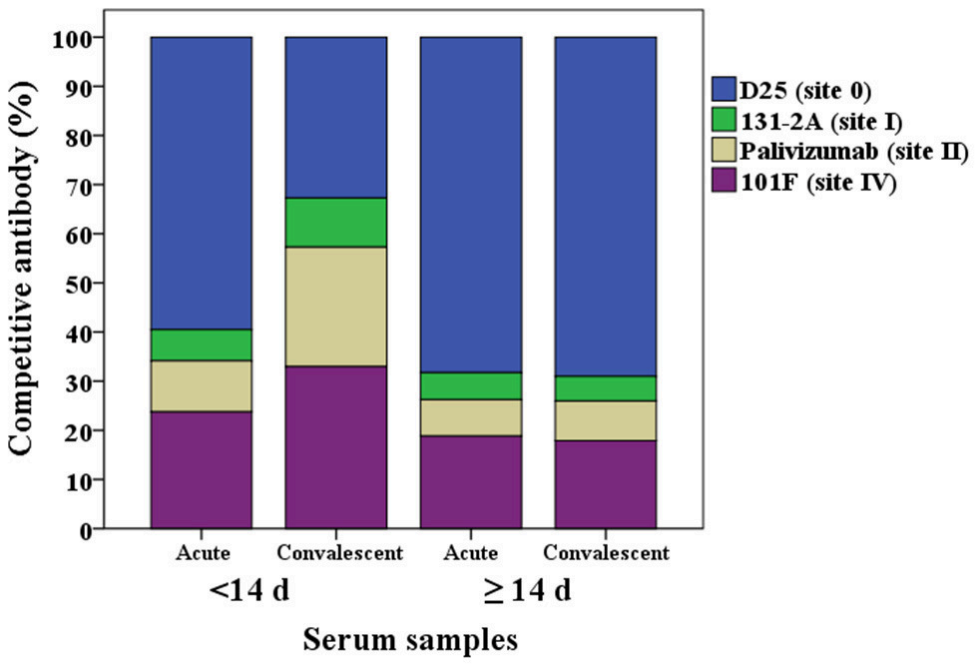
Percentage of each RSV antigenic site-specific competitive antibody GMC (μg/mL) to the total GMC (μg/mL) for acute and convalescent sera from HCT adults shedding virus <14 days and ≥14 days. The Y axis is the percentage of each competitive antibody GMC to the total GMC. The X axis is the serum types: acute or convalescent sera from HCT adults shedding virus <14 days or ≥14 days. The total GMC was the sum of 4 competitive antibody GMC. The percentage of each competitive antibody was that the competitive antibody GMC divided by the total GMC. GMC, geometric mean concentration. *N* = 40 in each competitive antibody assay.

The proportion of the contribution for each site-specific competitive antibody GMC to the total GMC for acute and convalescent sera from HCT adults shedding virus <14 days and ≥14 days are illustrated in Figure 3. For HCT adults shedding virus <14 days, ~60% of the total GMC was composed by D25-competing antibody, and decreased to ~30% in the convalescent sera; while the percentages of 131-2A, palivizumab-competing antibody, and 101F-competing antibodies increased. For HCT adults shedding virus ≥14 days, ~70% of the total GMC was also composed by D25-competing antibody in the acute sera, and the percent contribution by the four competitive antibodies did not increase even though the total GMC increased from 34.1 μg/mL in the acute sera to 91.8 μg/mL in the convalescent sera.

### No Significant Differences Were Observed in Antibody Levels in the Acute and Convalescent Sera Between HCT Adults Infected With RSV/A vs. RSV/B

The site-specific competitive antibody GMC and neutralizing antibody GMT were compared between RSV/A and RSV/B infected HCT adults (Table 3). No significant differences were observed in both the neutralizing antibody level and site-specific competitive antibody concentrations in the acute and convalescent sera between HCT adults infected with RSV/A vs. RSV/B. An increase in antibody activity were detected in the convalescent sera of RSV/A and RSV/B infected HCT adults by all 6 assays. In addition, the total GMC of the 4 competitive antibodies were comparable (133.6 vs. 159.9 μg/mL) in convalescent sera between RSV/A and RSV/B infected HCT adults. This observation is consistent with antigenic site II and IV being well conserved among RSV isolates in the RSV/A and RSV/B subgroups.

**Table 3:** RSV Antibody Levels between RSV/A and RSV/B Infected HCT Adults.

**RSV antibody**	**RSV antibody target**	**Serum**	**RSV/A infected adults (*n* = 22)**	**RSV/B infected adults (*n* = 18)**	***P*-value^b^**
Competitive antibody	Ø	Acute	23.0 [16.1, 42.5]^a^	20.3 [12.9, 31.8]	0.423
		Convalescent	50.0 [27.6, 86.5]	62.1 [39.0, 92.1]	0.570
	I	Acute	2.5 [1.4, 5.1]	1.5 [0.9, 2.4]	0.213
		Convalescent	8.7 [3.5, 21.7]	7.6 [3.5, 15.9]	0.814
	II	Acute	3.7 [1.9, 7.5]	2.1 [1.4, 3.2]	0.176
		Convalescent	14.9 [6.0, 36.7]	18.1 [7.5, 43.7]	0.754
	IV	Acute	8.2 [5.2, 14.4]	5.8 [3.9, 8.8]	0.339
		Convalescent	25.7 [12.2, 60.8]	31.5 [16.0, 62.4]	0.678
	Ø, I, II, IV	Total Acute	45.0 [28.8, 75.1]	35.1 [25.0, 49.5]	0.442
		Total Convalescent	133.6 [72.7, 234.1]	159.9 [104.4, 256.1]	0.640
Neutralizing antibody	RSV/A	Acute	6.7 [5.6, 7.8]	6.9 [5.7, 8.1]	0.510
		Convalescent	9.1 [8.1, 10.3]	8.9 [7.5, 10.4]	0.807
	RSV/B	Acute	6.5 [5.7, 7.2]	6.7 [6.0, 7.7]	0.593
		Convalescent	9.3 [8.0, 10.7]	9.8 [8.3, 11.6]	0.395

a *Geometric mean conc., μg/mL for competitive antibody and log2 for neutralizing antibody titer [95% Confidence Interval]*.

b *Two-sample t-test for difference in means of antigenic site competitive Ab conc. (log2 μg/mL) or neutralizing antibody titer (log2) between RSV/A and RSV/B*.

**Figure 4:**
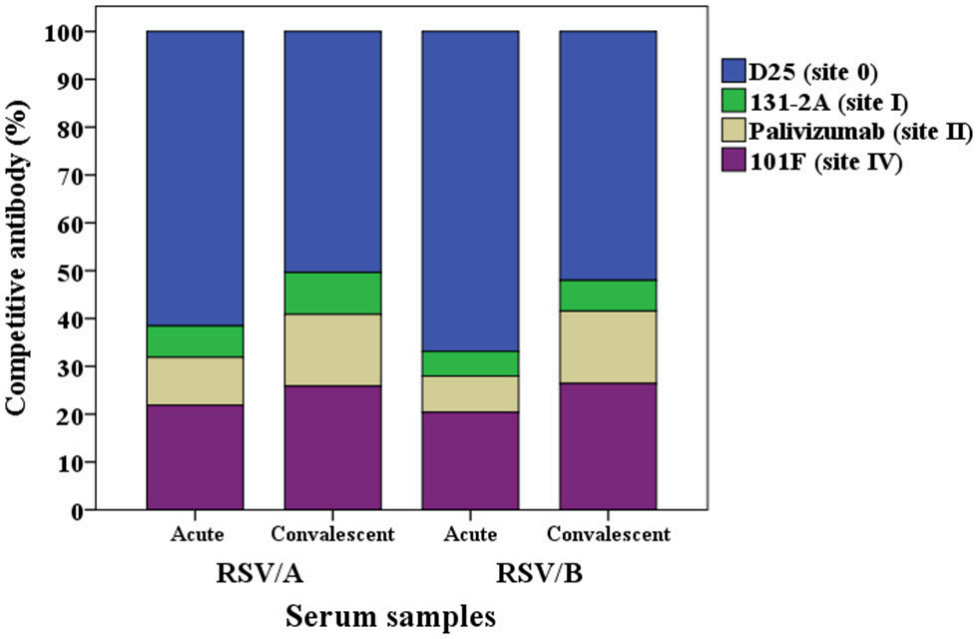
Percentage of each RSV site-specific competitive antibody GMC (μg/mL) to the total GMC (μg/mL) for RSV/A and B infected HCT adults. GMC, geometric mean concentration. *n* = 40 in each competitive antibody assay.

The proportion of the contribution for each site-specific competitive antibody GMC to the total GMC for RSV/A and B infected HCT adults is illustrated in Figure 4. D25-competing antibody constitutes ~60% of the total GMC in the acute sera for both RSVA and B infected HCT adults, and decreased to ~50% in the convalescent sera; while the percentages of 131-2A, palivizumab-competing antibody, and 101F-competing antibodies increased in the convalescent sera for both A and B infected HCT adults.

### Site-Specific Competing Antibodies Correlate to RSV-Specific Neutralizing Antibodies

**Figure 5:**
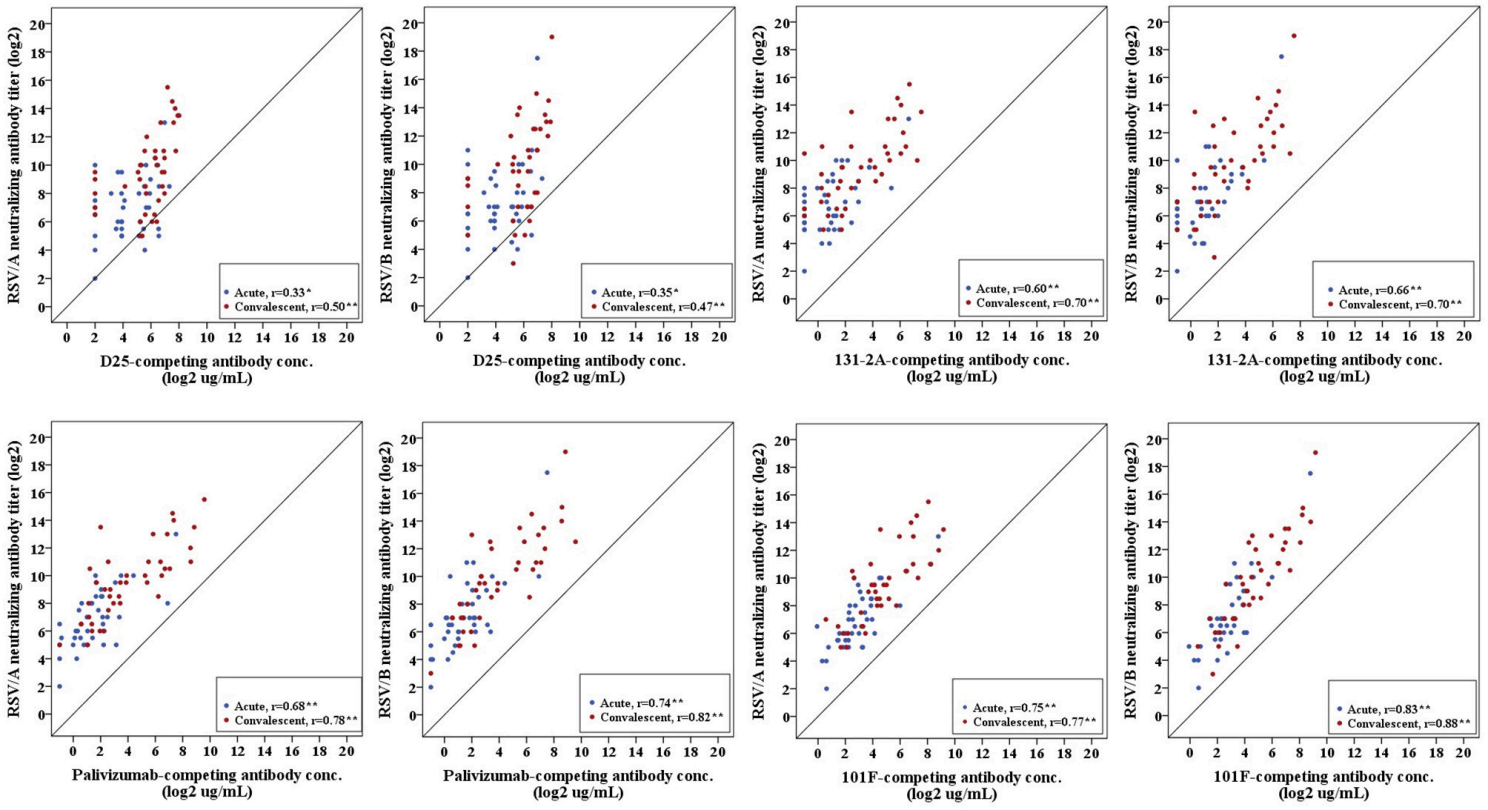
Correlation of RSV antigenic site-specific competitive antibody and RSV-specific neutralizing antibody. Pearson's correlation coefficient was calculated to measure the strength of the linear association. Correlation coefficients ranged from 0.33 to 0.83 for acute sera, and 0.50–0.88 for convalescent sera. ^*^Correlation is significant at the 0.05 level (2-tailed). ^**^Correlation is significant at the 0.01 level (2-tailed). *n* = 40 in each competitive antibody assay and microneutralization assay.

Pearson's correlation coefficient was calculated to measure the strength of the linear association between site-specific competitive antibodies and RSV-specific neutralizing antibodies. We observed a significant positive correlation between site-specific competitive antibody measured by the four site-specific competitive antibody assays to neutralizing antibody measured by the RSV/A and RSV/B microneutralization assays. Correlation coefficients ranged from 0.33 to 0.83 for acute sera, and 0.50 to 0.88 for convalescent sera with all correlations being significant (*p* < 0.05) (Figure 5). The highest correlation was observed between the site IV 101F-competing antibody concentration and RSV neutralizing antibody titers, and the lowest correlation was observed between site Ø D25-competing antibody concentration and RSV neutralizing antibody titers in RSV infected HCT adults.

## Discussion

An in-depth understanding of the human antibody response to RSV infection will aid the development and evaluation of vaccines and therapeutics against RSV disease. Previous studies have reported on the epitopes targeted by RSV-specific neutralizing antibodies in human sera (35, 36), as well as reported the specificities and functional properties of antibodies elicited by natural RSV infection (17). To our knowledge, the present study is the first to describe site-specific competitive antibody responses to the pre-F and post-F conformations and to correlate these site-specific competitive antibody responses to viral clearance and neutralizing antibody responses in RSV naturally infected HCT adults.

In this prospective cohort study, pre-F and post-F competitive antibodies were elicited after RSV infection in HCT adults. The highest site-specific competitive antibody concentration in acute sera was against site Ø on the pre-F conformation, which is consistent with prior published results showing that prefusion F-specific antibodies are prevalent in polyclonal neutralizing antisera (36). However, the high levels of site Ø specific competitive antibodies present in the ≥14 days group were ineffective in viral clearance once virus infection was established. Ngwuta et al. (36) used methods that relied on absorption to define the neutralizing activity associated with pre-F and post F conformations as well as site Ø and site II. Our site-specific competitive antibody assays measured site-specific competitive antibody concentrations but did not directly demonstrate the neutralizing activity of the antigenic site-specific competing-antibodies. In general, RSV-specific antibodies play an important role in preventing infection and a lesser role in viral clearance. The group that cleared the virus in <14 days were able to generate a more vigorous humoral response likely reflecting greater immunologic reconstitution after stem cell transplantation. Although we did not measured cell mediated immunity, it is highly likely that this group also had a more robust cellular immune response that contributed to improved viral clearance.

Site I had the lowest competitive antibody concentration, which is also consistent with the results published by Gilman and others (17, 37). The highest fold increase was detected against site II, suggesting that following RSV infection the immune response recognizes other antigenic sites that are not unique to the pre-F form. This was demonstrated by the increase in the proportion of site II, site IV and site I competitive antibody GMC but not site Ø to the total GMC in the convalescent sera of HCT adults who rapidly cleared their viral infection. It is not clear why antibodies that target sites unique to the pre-F form appear to persist for longer duration compared to sites shared by the pre-F and post-F forms. It is important to point out that most of the competitive antibodies were directed to site Ø in the acute serum samples. If similar concentration increases occurred to the four antigenic sites in the convalescent serum samples, this would have resulted in higher proportion increase of site-specific competitive antibodies that had lower concentration in the acute serum samples such as sites II and IV. The change in proportion in site-specific competitive antibody was compounded by a higher LLoD (7.8 μg/mL) for the cell based site Ø competitive antibody assay that was appreciably higher compared to the LLoD (1.0 μg/mL) for the other three competitive antibody assays.

We used Pearson's correlation to determine the associations between the four site-specific competitive antibodies and between site-specific antibody concentrations to neutralizing antibody titers. The highest correlations to RSV-specific neutralizing antibody levels were observed with sites II and IV competitive antibodies. These results suggest that antibodies against palivizumab and 101F epitopes are responsible for a significant proportion of the virus neutralizing capacity of sera. It also suggest that the site-specific competitive antibody assays measures changes that directly relates to changes in neutralizing antibody levels. The lower correlation detected between antigenic-site Ø competitive antibody concentrations and neutralizing antibody titers might be related to antigenic site Ø being the least conserved region compared to other antigenic sites in the F protein (13, 38, 39), although this was not analyzed in the present study. The low correlation also might be due to the relative high LLoD of the site Ø antibody competitive assay compared to the lower LLoD for the other three site-specific competitive antibody assays. Additionally, small changes in antigenic site Ø competitive antibody concentration might be associated with greater changes in neutralizing antibody activity, thereby making it harder to detect a strong correlation due to assay variability. Of the four mAbs we tested for neutralizing antibody activity, D25 mAb that targets site Ø was the most potent of the mAbs, followed by 101F and palivizumab; 131-2A shows no neutralizing potency, which were consistent with what Phung and others reported (40). These different neutralizing antibody activities suggest that sites Ø, II, and IV are major target of the human neutralizing antibody response, and antigenic site I is not a major target. Interesting, although there was a strong correlation between site I competitive antibody concentration and RSV-specific neutralizing antibody titers, the site I mAb (131-2A) did not have detectable neutralizing antibody activity. The direct correlation observed likely reflects the HCT adults' ability to mount an effective humoral response to antigenic sites on the F protein rather than a mechanistic correlation between site I competitive antibody concentration and neutralizing antibody activity.

The effect of RSV infection by viral subgroups (RSV/A and RSV/B) on the humoral immune responses to the four antigenic sites was evaluated in this prospective cohort study. No significant differences were observed in both the neutralizing antibody level and site-specific competitive antibody concentrations measured between HCT recipients infected with RSV/A vs. RSV/B. In addition, the proportion and the total GMC of the four competitive antibodies were comparable in convalescent sera between RSV/A and RSV/B infected HCT adults. This observation is consistent with antigenic site II and site IV being well conserved between RSV/A and RSV/B subgroups, and suggests a monovalent RSV-F vaccine against infection with RSV/A and RSV/B is likely sufficient for protection against severe RSV disease.

Our study has some limitations. The small number of RSV infected HCT adults (*n* = 40) is not representative of adults in the general population. Higher rises in competitive antibody concentration and neutralizing antibody titer after an RSV infection might occur in healthy adults compared to the HCT adults. In addition, we did not measure competitive antibody concentration to site III and site V, the remaining pre-F antigenic sites. Thus, their contribution to the neutralizing antibody activity post-RSV infection was not determined in this cohort of HCT adults infected with RSV.

In summary, the study revealed significantly higher concentrations in RSV F site-specific competitive antibodies (except for site Ø) in HCT adults who shed RSV <14 days compared to ≥14 days, comparable concentrations of competitive antibodies in the convalescent sera between RSV/A and RSV/B infected HCT adults, and a significant positive correlation between site-specific competitive antibodies and RSV-specific neutralizing antibody activity. In conclusion, in RSV naturally infected HCT adults, site-specific competitive antibody responses occurred to antigenic sites found in both the pre-fusion and post-fusion F conformations and were associated with viral clearance. The data might suggest but does not demonstrate that these antibody responses are contributing to the observed more rapid viral clearance. The faster clearance in the individuals with higher antibody responses might just reflect the level of immune competence of these individuals. Therefore, the observed relation between higher antibody responses and more rapid clearance is not necessarily the cause and the consequence. In addition, further evaluation of how the T cell–mediated responses assist in RSV viral clearance is needed. Lastly, HCT recipients would likely benefit from monoclonal antibody immunoprophylaxis or RSV-F vaccine when licensed for prevention of severe RSV infection during their period of vulnerability post-transplantation.

## Data Availability

The datasets generated for this study are available on request to the corresponding author.

## Ethics Statement

This study was carried out in accordance with the recommendations of the institutional review boards of the University of Texas MD Anderson Cancer Center and Baylor College of Medicine with written informed consent from all subjects. All subjects gave written informed consent in accordance with the Declaration of Helsinki. The protocol was approved by the University of Texas MD Anderson Cancer Center and Baylor College of Medicine.

## Author Contributions

XY, RC, and PP designed the research. XY, OI, VA, LA, TM, and PP performed the research. XY, LF-S, and PP analyzed the data. DS and RC provided the samples. KP processed the samples. F-AP, VA, LA, TM, LF-S, KP, DS, RC, and PP edited the text. XY wrote the first draft of the manuscript.

### Conflict of Interest Statement

The authors declare that the research was conducted in the absence of any commercial or financial relationships that could be construed as a potential conflict of interest.
